# Informing relatives of non-surviving patients about research participation following out-of-hospital cardiac arrest: a prospective cohort study

**DOI:** 10.1016/j.resplu.2025.101131

**Published:** 2025-10-15

**Authors:** Helen Pocock, Charles D. Deakin, Ranjit Lall, Tom Quinn, Nigel Rees, Isabel Rodriguez-Bachiller, Deb Smith, Gavin D. Perkins

**Affiliations:** a South Central Ambulance NHS Foundation Trust, Bicester, UK; b Warwick Clinical Trials Unit, Warwick Medical School, University of Warwick, Coventry, UK; c University Hospitals Southampton NHS Foundation Trust, Tremona Road, Southampton, Hampshire, UK; d Kingston University, London, UK; e Welsh Ambulance Service University NHS Trust, Swansea, UK; f University Hospitals Birmingham NHS Foundation Trust, Birmingham, UK

**Keywords:** Out-of-hospital cardiac arrest, Resuscitation, Study notification, Cohort study

## Abstract

**Background:**

Currently, it is not common UK practice to actively inform relatives of non-surviving patients about enrolment in out-of-hospital cardiac arrest (OHCA) trials. However, in the US and elsewhere, researchers routinely notify relatives at the earliest opportunity. We assessed the process of writing to relatives of non-surviving patients enrolled in the Prehospital Optimal Shock Energy for Defibrillation (POSED) study.

**Methods:**

This prospective, non-randomised cohort study was a sub-study of the POSED randomised controlled trial. Together with public and patient partners, we developed a condolence letter, minimising the burden of information whilst offering support and further information. Next of kin contact details were verified using two independent sources. Acceptability was assessed by monitoring enquiries or complaints received. We recorded time taken to gather details, discordance between sources and number of cases where we could not obtain details.

**Results:**

We sent letters to 18/33 (55 %) of relatives. Fifteen letters were not sent because we could not obtain relative’s details (n = 5), could not verify with another source (n = 3), found discordance between sources (n = 5), or the agreed time window had passed (n = 2). The median time spent per case was 27 min (IQR 20–40). No enquiries or complaints were received.

**Conclusions:**

Identifying the next of kin for individuals who did not survive OHCA was challenging and required significant resources. However, no comments or complaints were received from relatives who were sent information about trial participation.

**Trial registration number:**

ISRCTN16327029

## Highlights

• Bereaved relatives are not routinely informed of trial enrolment in UK cardiac arrest trials.
• This study assessed sending condolence letters to relatives in the POSED trial.
• We sent 18 of 33 letters; contact or timing issues precluded sending others.
• No complaints or enquiries were received from recipients.
• The approach was viable but requires streamlining for wider implementation.

## Background

Clinical trials comparing cardiac arrest treatments, by their very nature, can only be conducted in patients who lack capacity at the time of treatment. At best, they will find out about their research enrolment after the event. In England, only 9.5 % of patients receiving CPR survive to hospital discharge,^1^ and globally only 10.7 % of patients survive to one month.^2^ When an enrolled patient dies before being informed about research participation, the decision regarding whether to inform their relatives varies across studies, but is based on weighing up transparency against possible harm from the emotional distress that might be caused.^3^ Bereaved relatives may be under extreme psychological burden at this time due to the sudden change in family dynamics and roles.^4^

Previous UK studies have not actively informed relatives of the patients’ research inclusion; an approach supported by patients and public alike.^5^, ^6^ Patients and public advisors have instead supported either providing no information or providing it via passive means. Passive mechanisms may include posters displaying general study messages, empowering relatives to choose whether to seek specific information. However, some public partners in the Prehospital Optimal Shock Energy for Defibrillation (POSED) study advisory group challenged this approach.^7^ Their concerns were that ‘no information’ or ‘passive information’ approaches lacked openness and transparency. By not actively providing study information, there was a risk that relatives either would not find out at all or would find out through other channels (e.g., at an inquest).^8^ The active approach to informing the relatives of non-survivors had not previously been tested in the UK. It is, however, a common approach elsewhere.

In the USA, for both drug and device trials where permission for exception or waiver of informed consent is obtained, there is a legal requirement for researchers to attempt to notify relatives of the trial enrolment at the earliest opportunity.^9^ This is usually communicated via letter and appears to have been acceptable to recipients.^10^, ^11^, ^12^ However, the UK is not an outlier in not actively informing relatives. A recent international survey revealed that between 2010 and 2022, cardiac arrest researchers actively notified relatives in 44 % of studies, 39 % provided no information and 17 % provided information passively.^13^ Where information was not actively provided, this was often because researchers felt it was not appropriate, or their community consultation indicated opposition.^13^ Community consultation was central to the present study, informing each step of its development and delivery. The feasibility nature of the POSED study provided an opportunity to test new approaches to trial delivery.

The aim of this sub-study was to explore the process of actively informing relatives of non-survivors enrolled in the POSED study. This cohort study has been reported in line with the STROCSS guidelines.^14^

## Methods

### Study design

This prospective, non-randomised cohort study was a sub-study of the Prehospital Optimal Shock Energy for Defibrillation (POSED) randomised controlled trial.^15^ Patients were enrolled into the POSED study between March 2022 and February 2023 by paramedic teams of the South Central Ambulance Service NHS Foundation Trust, which serves four counties in the south of England. The study was approved by the London (Harrow) NHS Research Ethics Committee (20/LO/1242) and prospectively registered on the ISRCTN Trial Registry (ISRCTN16327029; https://doi.org/10.1186/ISRCTN16327029).

The protocol has been described previously but, in brief, adult patients were enrolled if they underwent a resuscitation attempt and were treated by the participating ambulance service with a shock from a study defibrillator.^7^ Patients were followed up for 30 days. Surviving patients were informed of their study enrolment face to face when the initial emergency phase was over. This occurred when the study team received confirmation from the hospital that the patient had been transferred from critical care to a general ward (median 25 days post arrest). This study explores the attempts of researchers to notify relatives of non-surviving patients by letter of their study enrolment.

### Patient and public involvement

We considered the suitability of informing relatives together with a panel comprising a relative of a cardiac arrest survivor, three people with previous training in defibrillator use (of whom two were community lay responders), one recipient of NHS cardiac care and one person with prior experience of representing patient and public views.

Opinions were divided. Some contributors felt delivering news of a study enrolment might introduce an additional and unnecessary emotional burden. Others felt that relatives might derive some comfort from knowing that their relative’s death resulted in a positive contribution to research. To not inform them would be to deny them this comfort and might be considered paternalistic.^16^ There are ethical challenges with either approach. Given the choice, relatives might not want to know, and by actively approaching relatives to deliver this news, the act of choosing was precluded. A passive approach was suggested; however, this would not guarantee reaching relatives of every patient and discovery of the enrolment later may result in greater emotional distress and loss of trust in the NHS.

On balance, the patient partners felt that active and early provision of information would cause less harm than finding out later, which may set people back in the grieving process. It was suggested that news of research enrolment need not be frightening if presented in the right way and was decided that a sensitively worded letter would be the vehicle for actively informing the relatives of non-surviving patients.

We co-developed a letter with the study panel. There was a lack of consensus regarding the timing of delivery of the letter, with public partners advocating for anywhere between four weeks and six months. Some felt that a letter should not be presented too soon as relatives may be unable to take in information at such a time of distress, whilst others cautioned against delay for fear of reopening healing emotional wounds. There did not seem to be any specific commonality, in terms of demographics or experience, between those advocating for either approach.

Since opinions were divided, further patient and public input was sought from the University Hospitals Birmingham NHS Foundation Trust Clinical Research Ambassador Group, and a patient representative from a local hospital emergency department (ED), two of whom had lost relatives to cardiac arrest. Additionally, we contacted international researchers and the bereavement lead at a local hospital to learn of their experiences.

### Participant recruitment

We monitored survival status of patients in the POSED study between Day 0 and Day 30. Hospital research teams were contacted for status updates of patients conveyed to hospital. Surviving patients and/or their families were approached in hospital once the patient had been moved out of critical care and onto a ward. For this sub-study, next of kin of non-surviving patients who died before 30 days (prehospital or in-hospital), who were not already notified of their enrolment, were eligible for inclusion. As soon as we were aware of a patient’s death, we attempted to source next of kin contact details from EMS clinical notes, NHS Summary Care Records or other community medical provider records. If next of kin could be identified and verified, a letter was prepared. It was sent within the four to six week post death time window. Survival status of patients enrolled in the POSED study was monitored from 3rd April 2022 to 16th Feb 2023; the time window for sending letters was 1st May 2022 to 30th March 2023.

### Intervention

We created a letter of condolence that minimised the burden of information whilst offering the opportunity of support and further information if desired. We felt that this approach empowered people to decide whether and when to seek information. In addition to our public partners, international researchers, and a bereavement lead, the study Sponsorship committee, the NHS ethics committee and the local EMS advised on, and approved, wording of the letter. A national cardiac arrest trial was running concurrently with the POSED study. We co-enrolled patients into the PARAMEDIC3 trial where appropriate, a strategy acceptable to patient partners of both trials. Relatives of co-enrolled patients were informed of their enrolment into both studies via an adapted version of the letter. Both letters are provided in the Supplementary Materials. Communication with relatives was solely by letter. The panel emphasised the need to make best efforts to reach the patient’s nominated next of kin, as identified by the patient, to reduce the risk of disclosing sensitive information to an unintended recipient.

Based on the practices of international researchers and that of local hospital bereavement services, we agreed that delivery of the letter four to six weeks post death would be optimal. At this time the immediate intense grieving period might be over but not yet resolved, such that a letter would be unlikely to ‘reopen old wounds’.

We sought recipients’ details from the Ambulance Service electronic patient record (ePR), the NHS Summary Care Record and/or via phone call or email to the patient’s General Practitioner (GP) surgery. Concordance of two data sources was required to avoid disclosing sensitive information to other parties.

### Outcomes

Acceptability of the approach to informing relatives was assessed throughout the study by monitoring the number and nature of enquiries received by the EMS provider. We noted the time taken to identify next of kin (including number of phone calls/emails) and number of sources consulted to verify their details. Concordance/discordance between the different sources was recorded as was the number of cases where it was not possible to identify next of kin.

## Results

### Recruitment

**Fig. 1:**
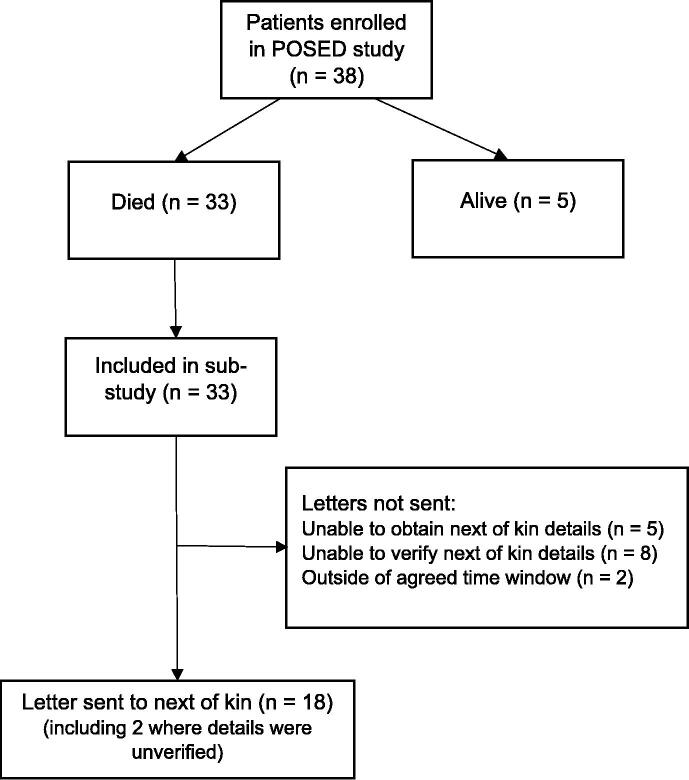
Flow chart of patient enrolment.

Between 22nd March 2022 and 28th February 2023, the POSED study enrolled 38 patients with OHCA of whom 33 (87 %) died during the time window for this study and were eligible for inclusion in this study (Fig. 1).

### Next of kin identification and checking process

Initially, confirmation was sought that the patients had not registered with the NHS National Data Opt-Out (NDOO) system, a scheme whereby patients may register their wish for their personal health data not to be used for research. Next of kin details were sought for the 31/33 patients for whom no NDOO was registered. Relatives were identified from the ambulance electronic patient record (ePR) (26/31 next of kin details were obtained from this source).

**Fig. 2:**
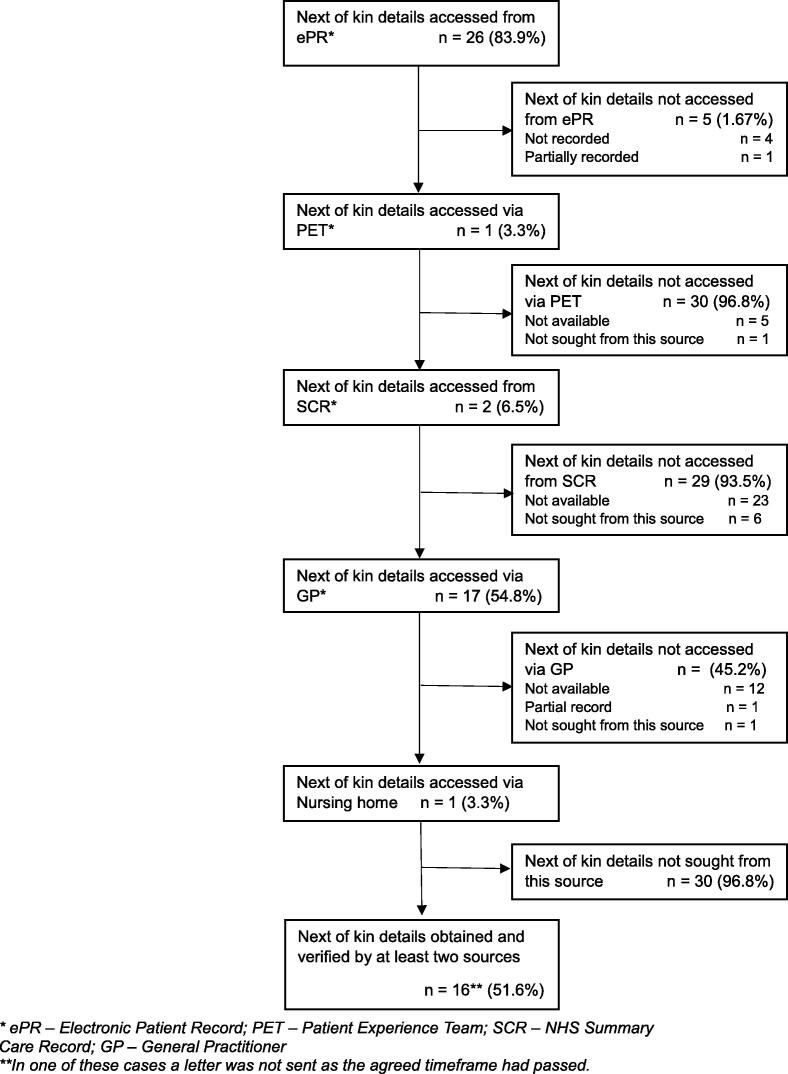
Sources consulted for next of kin details.

Verification was attempted initially via the EMS Patient Experience Team (PET) (5/31 next of kin details were provided), but we later consulted the NHS Summary Care Records (SCR) system directly (2/31 details located). GP surgeries were the source of next of kin details in 17/31 cases and a Nursing Home provided contact details in 1/31 case. Fig. 2 shows the sources consulted and success rates. Table 1 summarises the resource requirement to attempt to verify patients’ next of kin. Further details are provided in the Supplementary Materials. It does not take account of checks (and double checks) of the patients’ ambulance service electronic records.

**Table 1:** Summary of resource requirements and outcomes for verification of next of kin details.

	**Total (n, unless specified)**
Emails sent	15
Phone calls made	25
Mean time spent	31.7 min (range 10–65)
Concordant cases (verified by two or more sources)	15
Discordant cases (details not aligned between sources)	5
Unverified cases (not recorded on more than one source)	9
Details unobtainable (not recorded on any source)	2

We were able to identify and send letters to the relatives of only 18 (55 %) of the non-surviving patients. Fifteen letters could not be sent because either next of kin details could not be obtained (n = 5) or could not be verified (n = 10). Letters were not sent in two cases as the agreed time window had passed This was due to delays in establishing whether the patient was registered with the NHS National Data Opt-Out service. In five cases there was discordance between different sources of next of kin details. In two of these cases, letters were sent (prior to a Study Management Group decision not to send letters in cases of discordance).

### Resource considerations

In total, researchers spent 16 h administering the letters. A median 27 min (IQR 20–40 min) was spent attempting to contact the relatives of each non-surviving patient. The current UK postage cost per letter is £1.70 ($2.28 US).

### Response to letters received

No enquiries about the study or complaints were received by the Ambulance Service. No letters were returned unopened.

## Discussion

This study reports on the success of a strategy, used for the first time in the UK, to inform next of kin of non-surviving patient of enrolment in an out-of-hospital cardiac arrest study. We were able to send letters to the next of kin of 55 % of patients. None of the information sources consulted consistently collected next of kin contact details. The resource requirement for researchers and EMS was considerable at a mean 32 min for each case.

The acceptability of our notification approach is open to various interpretations. Our primary measure of acceptability was the recipients’ response to the letters. Using the number of complaints received as a surrogate indicator of acceptance, it could be inferred that we found 100 % acceptability. However, only by seeking the opinions of the recipients could this claim be made with validity. North American studies have also employed a similar approach to assessing acceptability, using the rate and nature of responses from relatives as a proxy. In one study, 12.9 % of relatives made contact, with comments or questions in response to a letter but none were negative and only 0.3 % of patients were withdrawn from follow-up.^11^ In another, just 0.3 % of those who contacting researchers expressed negative feedback and 0.91 % of patients were withdrawn.^10^ These findings, alongside our own, suggest that both emergency research conducted without prior consent and a carefully managed notification process may be broadly acceptable to the public.

The other aspect to acceptability is whether this activity is acceptable to researchers and funders. A significant proportion of relatives could not be contacted since we could not obtain or verify their contact details. This makes the strategy unreliable and the current inability to triangulate next of kin information would likely make researchers, funders and research ethics committees reluctant to support this strategy. Provision of information to only 55 % of relatives is an unsatisfactory outcome for this strategy. The inability to confidently establish contact details for relatives in 45 % of cases was much higher than expected. North American studies have reported being unable to ascertain contact information for relatives of between 0.5 % and 11.5 % of patients^8^, ^9^, ^10^ although it would have been possible to increase the number of letters sent by omitting the verification check. In a previous study where 95 % of contact details were obtained, 9 % of letters were undeliverable/returned to sender^10^ highlighting the importance of triangulating contact information. The EMS electronic patient record (ePR) contains a data field for next of kin (name/relationship) but does not include an address field. Even where this information was available, discordance with other sources was noted in 17 % of cases.

The time and resource commitment to achieve only 55 % activity completion is not inconsiderable. Scaled up for a main trial of 3462 patients, around 1846 h (49-weeks) would need to be dedicated to this activity. Accounting for annual leave, this would represent over a year of full-time work for an administrator, at a cost of almost £30,000 according to current NHS pay scales. A more reliable and efficient means of obtaining and verifying next of kin details would be required to make this strategy cost-effective.

There is much variability across the cardiac arrest research community regarding the approach to informing the relatives of non-survivors of research participation.^12^ Researchers reported a variety of influences on their chosen strategy. The active provision of information was mandated in law in the North American studies,^9^, ^10^ whereas in the absence of legal guidance, UK law does not mandate the approach taken, placing the onus on researchers to make an empirical decision for their studies. Passive methods such as posters have been used previously although, like other media campaigns, they offer no guarantee of reaching the intended audience. For example, only 46.7 % of survey respondents in Wales recalled recent campaigns on violence and aggression towards ambulance staff,^17^ and just 5 % of patients noticed research posters in Emergency Department waiting rooms.^18^ Additionally, passive notification can leave researchers vulnerable to inaccurate or uncontrolled information sharing.^19^ A current UK study of in-hospital cardiac arrest decided their approach in consultation with patient and public advisors and the research ethics committee.^20^ The avoidance of confusion and distress to relatives, coupled with the fact that the treatments compared were in current use and had similar equipoise, was the rationale for not informing relatives.^18^ This suggests that whilst researchers are sensitive to the normative influence of the research culture within which they practice, they also proactively seek the best approach by consulting with public partners. Cardiac arrest researchers typically report no adverse consequences resulting from information provision whatever the means of delivery. We opted to contact next of kin via letter, but other methods, including telephone calls and face to face meetings, are commonly used.^13^ This decision was informed by our public partners, who advised that a letter would be less intrusive than a phone call or visit, and would offer recipients greater autonomy in deciding whether or not to seek further information about the study.

In the absence of any complaints in the current study, the letter developed could act as a template for future studies.

A key strength of this study was its foundation in input from public partners and inclusion of diverse stakeholder perspectives. We also systematically recorded the sources of relatives’ contact details and resource requirements for gathering this information. There were several limitations. Firstly, there was a very small sample size (n = 33) and so results may be of limited generalisability. Linked to this is the limitation of context. To produce an ethical study, acceptable to the UK public in 2019–2023 does not necessarily mean that a main trial mirroring every element would go unchallenged in 2025 and beyond. Nor would the strategy necessarily be generalisable to other contexts and countries. The ethical decisions made during the research are, by necessity, time- and context-bound. Thirdly, an important missing piece of the picture is a qualitative investigation of the experiences of those receiving letters. This would help researchers decide whether it is a beneficial activity for future studies and the optimal timing and content (level of information) of letters. This is currently unexplored in the literature.^13^

## Conclusions

This study found that, within the UK context, although no complaints or other comments were received from recipients, it was challenging and resource intensive to identify the next of kin for people who did not survive an OHCA. Future research should explore methods for identification of next of kin and the acceptability of approaching relatives following enrol in cardiac arrest trials.

## Funding sources

Helen Pocock, funded by Health Education England (HEE)/10.13039/501100000272National Institute for Health and Care Research (NIHR) for this research project (Grant no. NIHR-ICA-CDRF-2018-04-ST2-005). The views expressed in this publication are those of the author(s) and not necessarily those of the NIHR, NHS or the UK Department of Health and Social Care. The funder had no input into in study design, collection, analysis or interpretation of data, writing of the report or the decision to submit the article for publication.

GDP is supported by the National Institute for Health Research (NIHR) Applied Research Collaboration (ARC) West Midlands. The views expressed are those of the author(s) and not necessarily those of the NIHR or the Department of Health and Social Care.

## Data sharing statement

No further data are available related to this sub-study.

## CRediT authorship contribution statement

**Helen Pocock:** Writing – original draft, Visualization, Project administration, Methodology, Investigation, Funding acquisition, Conceptualization. **Charles D. Deakin:** Writing – review & editing, Writing – original draft, Supervision, Methodology, Funding acquisition, Conceptualization. **Ranjit Lall:** Writing – review & editing, Writing – original draft, Supervision, Methodology, Funding acquisition, Formal analysis. **Tom Quinn:** Writing – review & editing, Methodology. **Nigel Rees:** Writing – review & editing, Methodology. **Isabel Rodriguez-Bachiller:** Writing – review & editing, Investigation. **Deb Smith:** Writing – review & editing, Methodology. **Gavin D. Perkins:** Writing – review & editing, Writing – original draft, Supervision, Methodology, Funding acquisition, Conceptualization.

## Declaration of competing interest

The authors declare the following financial interests/personal relationships which may be considered as potential competing interests: HP is a member of the International Liaison Committee on Resuscitation (ILCOR) Advanced Life Support task force committee. NR is a grant holder and in receipt of funding from NHIR and HCRW. NR is also a member of HCRW and NIHR funding panels. GDP is Editor for Resuscitation and Resuscitation Plus and holds grants from the NIHR, British Heart Foundation, Resuscitation Council UK (RCUK) and Laerdal. He is an employee of University of Warwick, University Hospital of Coventry and Warwick, University Hospital Birmingham and West Midlands Ambulance Service and holds Leadership roles with ILCOR, the European Resuscitation Council, and RCUK.
