# Supplementary material for: Informing relatives of non-surviving patients about research participation following out-of-hospital cardiac arrest: a prospective cohort study
Source: Resusc Plus. 2025 Oct 15;26:101131. doi: 10.1016/j.resplu.2025.101131 (PMC12605857; doi:10.1016/j.resplu.2025.101131)
Supplement: Supplementary Data 1 [file mmc1.docx]

**Supplemental materials**

**Letter to relatives**

To the family of <PATIENT NAME>

Dear <NoK NAME>,

It is with our deepest sympathies that we write to you today. We are very sorry for your loss and extend our condolences to you as you move through this period of shock and grief.

We are writing to inform you about one treatment that <PATIENT NAME> received from South Central Ambulance Service when <HE/SHE> sustained a cardiac arrest. In addition to receiving the best medical treatment currently recommended for out-of-hospital cardiac arrest <PATIENT NAME> was included in a research study aimed at finding the best defibrillator shock strength to treat this devastating condition. The optimal shock level is currently unknown and the three shock strategies being compared in this study are currently part of routine care in UK Ambulance Services. Further information about the research is enclosed with this letter as well as contact details if you have any questions.

Because this treatment, along with all other treatments, needed to be delivered immediately, <PATIENT NAME> was entered into the study without prior consent, in accordance with legal and ethical regulations that apply to research such as this.

South Central Ambulance Service is committed to continually improving care and outcomes for patients. We know that losing a family member is always difficult and we cannot make this situation feel better. What is learnt as a result of their involvement in this study will help patients in the future who are affected by this devastating condition.

If you wish to find out more about the research, you can contact the study team via <email address>. You are also welcome to contact the Patient Experience Team either by email at <email address> or <telephone number>.

We apologise for this intrusion and again offer you our deepest condolences.

Yours sincerely

<signature> <signature>

|  |
| --- |

*This project is funded by the National Institute for Health Research (project number NIHR-ICA-CDRF-2018-04-ST2-005)*

**Letter to relatives of co-enrolled patients**

To the family of <PATIENT NAME>

Dear <NoK NAME>,

It is with our deepest sympathies that we write to you today. We are very sorry for your loss and extend our condolences to you as you move through this period of shock and grief.

We are writing to inform you about one treatment that <PATIENT NAME> received from South Central Ambulance Service when <HE/SHE> sustained a cardiac arrest. In addition to receiving the best medical treatment currently recommended for out-of-hospital cardiac arrest <PATIENT NAME> was included in research aimed at finding the best defibrillator shock strength and the best method of medicine delivery to treat this devastating condition. The optimal shock level is currently unknown and it is not known whether it is more effective giving medicines into a vein or into a bone (bone marrow). The treatments being compared are currently part of routine care in UK Ambulance Services. Further information about the research is enclosed with this letter as well as contact details if you have any questions.

Because this treatment, along with all other treatments, needed to be delivered immediately, <PATIENT NAME> was entered into the study without prior consent, in accordance with legal and ethical regulations that apply to research such as this.

South Central Ambulance Service is committed to continually improving care and outcomes for patients. We know that losing a family member is always difficult and we cannot make this situation feel better. What is learnt as a result of their involvement in this study will help patients in the future who are affected by this devastating condition.

If you wish to find out more about the research, you can contact the study team via <email address>. You are also welcome to contact the Patient Experience Team either by email at <email address> or <telephone number>.

We apologise for this intrusion and again offer you our deepest condolences.

Yours sincerely

<signature> <signature>

|  |
| --- |

*This project is funded by the National Institute for Health Research (project number NIHR-ICA-CDRF-2018-04-ST2-005*

**Resource requirements for relatives’ letters**

| **Study number** | **Total time taken (min)** | **No. of emails** | **No. of phone calls** | **Sources checked** | **Required information provided?** | **Discordance/ Concordance** | **Letter sent?** |
| --- | --- | --- | --- | --- | --- | --- | --- |
| 001 | 27 | 0 | 1 | ePR | N | Concordance | Y |
|  |  |  |  | PET^a^ | N |  |  |
|  |  |  |  | GP | Y |  |  |
|  |  |  |  | Nursing home | Y |  |  |
| 002 | 27 | 0 | 1 | ePR | Y | Discordance | Y |
|  |  |  |  | PET | N |  |  |
|  |  |  |  | GP | Y |  |  |
| 003 | 57 | 0 | 2 | ePR | Y | Concordance | Y |
|  |  |  |  | PET | N |  |  |
|  |  |  |  | GP | Y |  |  |
| 004 | 27 | 0 | 1 | ePR | Y | Discordance | N |
|  |  |  |  | PET | N |  |  |
|  |  |  |  | GP | Y |  |  |
| 006 | 35 | 0 | 1 | ePR | Y | Discordance | Y |
|  |  |  |  | PET | N |  |  |
|  |  |  |  | GP | Y |  |  |
| 007 | 27 | 0 | 1 | ePR | Y | Concordance | N |
|  |  |  |  | PET | Y |  |  |
| 008 | 15 | 1 | 0 | ePR | N | None listed | N |
|  |  |  |  | SCR^b^ | N |  |  |
|  |  |  |  | GP | N |  |  |
| 009 | 15 | 1 | 0 | ePR | Y | Unverified | Y |
|  |  |  |  | SCR | N |  |  |
|  |  |  |  | GP | N |  |  |
| 010 | 65 | 0 | 1 | ePR | Y | Concordance | Y |
|  |  |  |  | SCR | N |  |  |
|  |  |  |  | GP | Y |  |  |
| 011 | 20 | 1 | 0 | ePR | Y | Concordance | Y |
|  |  |  |  | SCR | Y |  |  |
|  |  |  |  | GP | Y |  |  |
| 012 | 20 | 1 | 0 | ePR | Y | Concordance | Y |
|  |  |  |  | SCR | N |  |  |
|  |  |  |  | GP | Y |  |  |
| 013 | 20 | 1 | 0 | ePR | Y | Discordance | N |
|  |  |  |  | SCR | Y |  |  |
|  |  |  |  | GP | N |  |  |
| 014 | 50 | 0 | 1 | ePR | Y | Concordance | Y |
|  |  |  |  | SCR | N |  |  |
|  |  |  |  | GP | Y |  |  |
| 015 | 45 | 1 | 0 | ePR | Y | Discordance | N |
|  |  |  |  | SCR | N |  |  |
|  |  |  |  | GP | Partial |  |  |
| 019 | 45 | 1 | 1 | ePR | Y | Concordance | Y |
|  |  |  |  | SCR | N |  |  |
|  |  |  |  | GP | Y |  |  |
| 020 | 30.5 | 1 | 1 | ePR | N | Unverified | N |
|  |  |  |  | SCR | N |  |  |
|  |  |  |  | GP | N |  |  |
| 021 | 25 | 0 | 2 | ePR | Partial | Unverified | N |
|  |  |  |  | SCR | N |  |  |
|  |  |  |  | GP | N |  |  |
| 022 | 10 | 0 | 0 | ePR | N | None listed | N |
|  |  |  |  | SCR | N |  |  |
|  |  |  |  | GP | N |  |  |
| 023 | 17 | 0 | 1 | ePR | Y | Unverified | N |
|  |  |  |  | SCR | N |  |  |
|  |  |  |  | GP | N |  |  |
| 024 | 18.5 | 1 | 1 | ePR | Y | Unverified | Y |
|  |  |  |  | SCR | N |  |  |
|  |  |  |  | GP | N |  |  |
| 025 | 33 | 0 | 1 | ePR | Y | Concordance | Y |
|  |  |  |  | SCR | N |  |  |
|  |  |  |  | GP | Y |  |  |
| 026 | 27 | 0 | 2 | ePR | Y | Concordance | Y |
|  |  |  |  | SCR | N |  |  |
|  |  |  |  | GP | Y |  |  |
| 028 | 40 | 0 | 1 | ePR | Y | Concordance | Y |
|  |  |  |  | SCR | N |  |  |
|  |  |  |  | GP | Y |  |  |
| 030 | N/A | N/A | N/A | N/A | N/A | N/A (outside agreed timeframe) | N |
| 032 | 25 | 1 | 1 | ePR | Y | Unverified | N |
|  |  |  |  | SCR | N |  |  |
|  |  |  |  | GP | N |  |  |
| 033 | 50 | 1 | 1 | ePR | Y | Concordance | Y |
|  |  |  |  | SCR | N |  |  |
|  |  |  |  | GP | Y |  |  |
| 034 | 36 | 0 | 1 | ePR | Y | Concordance | Y |
|  |  |  |  | SCR | N |  |  |
|  |  |  |  | GP | Y |  |  |
| 036 | 50 | 1 | 1 | ePR | Y | Concordance | Y |
|  |  |  |  | SCR | N |  |  |
|  |  |  |  | GP | Y |  |  |
| 037 | N/A | N/A | N/A | N/A | N/A | N/A (outside agreed timeframe) | N |
| 038 | 30 | 1 | 1 | ePR | Y | Unverified | N |
|  |  |  |  | SCR | N |  |  |
|  |  |  |  | GP | N |  |  |
| 039 | 35 | 0 | 1 | ePR | Y | Concordance | Y |
|  |  |  |  | SCR | N |  |  |
|  |  |  |  | GP | Y |  |  |
| 040 | 40 | 1 | 0 | ePR | Y | Unverified | N |
|  |  |  |  | SCR | N |  |  |
|  |  |  |  | GP | N |  |  |
| 041 | 20 | 1 | 0 | ePR | Y | Unverified | N |
|  |  |  |  | SCR | N |  |  |
|  |  |  |  | GP | N |  |  |

*^a^ PET = Ambulance service Patient Experience Team*

*^b^ SCR = NHS Summary Care Record*
